# Promoting the Self-Regulation of Stress in Health Care Providers: An Internet-Based Intervention

**DOI:** 10.3389/fpsyg.2018.00838

**Published:** 2018-06-15

**Authors:** Peter M. Gollwitzer, Doris Mayer, Christine Frick, Gabriele Oettingen

**Affiliations:** ^1^Department of Psychology, New York University, New York, NY, United States; ^2^Department of Psychology, University of Konstanz, Konstanz, Germany; ^3^Department of Psychology, University of Hamburg, Hamburg, Germany

**Keywords:** stress coping, self-regulation, health care providers, mental contrasting, implementation Intentions

## Abstract

The aim of our internet-based intervention study was to find out whether healthcare professionals can autonomously down-regulate the stress they experience at their workplace, using an established self-regulation tool called Mental Contrasting with Implementation Intentions (MCII). Applying MCII to reduce stress implied for our participants to repeatedly engage in a mental exercise that (1) required specifying a wish related to reducing stress, (2) identifying and imagining its most desired positive outcome, (3) detecting and imagining the obstacle that holds them back, and (4) coming up with an if-then plan on how to overcome it. We recruited on-line nurses employed at various health institutions all over Germany, and randomly assigned participants to one of three groups. In the MCII group (*n* = 33), participants were taught how to use this exercise via email and the participants were asked to engage in the exercise on a daily basis for a period of 3 weeks. As compared to two control groups, one being a no-treatment control group (*n* = 35) and the other a modified MCII group (*n* = 32), our experimental MCII group showed a reduced stress level and an enhanced work engagement. We discuss the strengths and weaknesses of the present study as well as ways to intensify MCII effects on stress reduction.

## Introduction

There are numerous factors that can make the work of healthcare providers stressful. For instance, caregivers sometimes have to face the death of patients, and there can be conflicts with the family of the patient. They may also lack the necessary equipment to do a good job, but still be taxed with excessive responsibility for negative outcomes. The work load may simply be too high, and the cooperation with team members may be ineffective and unpleasant. Health care workers often feel untrained at the beginning of their career and not respected at the end. They often fail to grasp patients’ expectations about the intensity and quality of the care; this is also true for how satisfying the given care is experienced by the patients. The administrative mechanisms might be complex, and health care workers may not get the appreciation they deserve from their superiors. Moreover, many healthcare providers suffer a reduced quality and quantity of sleep which makes coping with the challenges listed even harder (for reviews see Tyler and Cushway, 1992; Lambert and Lambert, 2001; McVicar, 2003; Johnston et al., 2013).

Given these difficult work conditions, one wonders what kind of interventions might help healthcare professionals to reduce their stress. One intervention that is found to be effective in all kinds of work settings is teaching people to redirect attention away from incomplete work goals during their leisure time (Smit and Barber, 2016); another successful approach focuses on teaching people what determinants facilitate recovery from different types of job stress (e.g., control during off job-time) thus heightening respective self-efficacy feelings (Hahn et al., 2011). Further effective interventions aim at enhancing physical activity thus increasing sleep quality and quantity, as well as reducing lower back pain (e.g., Kwasnicka et al., 2017). Interventions that specifically help healthcare providers to distance themselves from upsetting events and negative experiences (i.e., to adopt a watch tower perspective) were found to buffer even highly anxious caregivers from short and longer-term emotional distress (Penner et al., 2016).

In all of these interventions, healthcare professionals were taught about specific behaviors that have been found to reduce stress (e.g., redirecting attention away from incomplete goals, control during off-job time, increasing physical activity, distancing from upsetting events) through individual coaching or in workshops with a group of participants. The relevant information was provided through presentations, in the form of brochures, or via the internet. Some of these interventions also stimulated the participants to think about when and where in everyday life they wanted to act on what they had been taught (e.g., Smit and Barber, 2016).

The intervention we tested in the present study is different to these approaches in many respects. In contrast to the previously described approaches, which taught participants to establish specific stress reduction behaviors (e.g., increased physical activity) and encouraged participants to use them, we left it up to the participants to identify their own personal idiosyncratic wish of less stress at the workplace. In addition, we asked them to identify and imagine the best outcome of wish fulfillment, and identify and imagine the most important personal obstacle that they anticipated with regard to wish fulfillment. Once participants detected their own personal obstacle they had to vividly imagine its occurrence. Thereafter they had to name an effective idiosyncratic behavior to surmount the obstacle, before making an if-then plan in the form of “if…obstacle, then I will… behavior to surmount obstacle.” Such a plan links the idiosyncratic obstacle (if-part), to the idiosyncratic behavior to overcome the obstacle (the then-part of the plan).

In sum, whereas in the stress reduction interventions used so far people are informed about an established way of coping with stress and encouraged to use it in order to reduce stress, the self-regulation strategy introduced in the present research focuses on heightening the autonomous control of stress reducing behavior through mental imagery. The imagery procedure is based on the integration of two established self-regulation strategies: mental contrasting and forming implementation intentions (e.g., Oettingen and Gollwitzer, 2010).

## Self-Regulation By Mental Contrasting (MC)

Mental contrasting triggers goal pursuit by juxtaposing positive future fantasies with obstacles of present reality. When people contrast their wishes of a positive future with the obstacles standing in its way, the energy needed to overcome these obstacles is activated, given that this obstacle can potentially be overcome. For example, a person might identify a wish related to stress reduction at the work place: “I would love to stay calm even when blamed by patients!” After this first step of wish identification, she would identify the best outcome of staying calm and imagine that outcome, such as herself responding to the patient in a comforting manner. She would then identify and imagine her obstacle: “my urge to tell patients’ off when they become unreasonable.”

Mental contrasting fosters behavior change across life domains: academic and professional achievement, interpersonal relationships, and health and wellbeing (reviews by Oettingen, 2012, 2014). Mental contrasting enabled students to learn foreign language vocabulary, improve in math, study abroad, and complete a vocational training. Mental contrasting also helped with finding integrative (win–win) solutions in negotiations and with good decision making in everyday life. In the social realm, mental contrasting has been found to strengthen interpersonal relations and lead to effective reconciliation. It also heightened tolerance, encouraged taking responsibility for members of disadvantaged groups, promoted help seeking in college students and help giving in emergency care nurses. It was even effective in enhancing physical activity in stressed out students who just started their college education (Ruissen et al., 2018).

Extensive experimental research on the underlying mechanisms of mental contrasting effects has demonstrated that mental contrasting is a conscious imagery strategy that affects non-conscious cognitive processes, motivation, and responses to feedback, which in turn facilitate wish fulfillment. Three cognitive processes taking place outside of awareness have been delineated as such mediators. First, mental contrasting induces people to interpret the present reality as an obstacle to wish fulfillment (e.g., a party is no longer a fun event but an obstacle to meeting one’ wish of getting a good grade in an upcoming exam; e.g., Kappes et al., 2013). At the same time, mental contrasting strengthens the implicit associative links between the desired future and the obstacle as well as between the obstacle and the instrumental behavior to overcome the obstacle (e.g., Kappes et al., 2012b; Kappes and Oettingen, 2014). With respect to motivation, a heightened energization level, as measured by systolic blood pressure and by subjective reports of energy, was found to mediate the effects of mental contrasting on a person’s efforts to realize her wishes (see e.g., Sevincer et al., 2014). And mental contrasting facilitates dealing with setbacks in a way that fosters resilience: It fosters the processing of information contained in setbacks, and it protects against a loss of subjective competence (Kappes et al., 2012a).

## Self-Regulation by Implementation Intentions (II)

Implementation intentions are if-then plans (review by Gollwitzer, 2014) in the following format: “If the critical situation X is encountered, then I will perform the goal-directed response Y!” These implementation intentions are to be differentiated from mere goal intentions. The latter merely specify desired end states (“I want to achieve goal X!” or “I want to exert behavior X!”). In implementation intentions, on the other hand, the if-component of an implementation intention specifies a future critical event or point in time, and the then-component specifies how one will respond once these situational cues are encountered. The person described above who fails to control her urge to tell patients off when they approach her with unreasonable complaints could make the following if-then plan: “And if I feel too weak to decisively put a halt to my urge, then I’ll tell myself: Just take a watchtower perspective and aim at being calm and constructive!”

Evidence that forming if-then plans enhances the rate of attaining desired outcomes and execute the respective instrumental responses have been obtained in many studies regarding achievement, health, sports, and social relationships (meta-analysis by Gollwitzer and Sheeran, 2006). Implementation intentions have been shown to be an effective strategy to overcome external distractions (e.g., an exciting video) or internal hindrances such as self-doubts (Thürmer et al., 2013) in the service of persistent action. Implementation intentions’ self-regulatory benefits for action control extend into emotion regulation as well (e.g., fear, disgust, and anger; summary by Webb et al., 2012). Finally, with respect to the regulation of cognitive responses research demonstrated that if-then plans help people to switch from reflexive to reflective thinking thus improving decision making (e.g., Bieleke et al., 2017).

But how do implementation intentions work? Experimental research found that the mental representation of the selected situation in the if-part becomes highly activated and hence more accessible (e.g., Webb and Sheeran, 2004). Moreover, linking the if-part to the then-part produces automaticity (Gollwitzer, 1999) in the sense that encountering the specified situation triggers the specified response in an automatic fashion: this response is now performed immediately, efficiently, and no further conscious intent is needed. Not surprisingly, then, various studies could show that people who form implementation intentions are in a good position to break unwanted habitual responses (e.g., Adriaanse et al., 2011).

## Mental Contrasting With Implementation Intentions (MCII)

Sometimes people face obstacles that are surmountable but are particularly hard to deal with (e.g., impulsive behavior, strong emotions, and ingrained habits). While mental contrasting builds non-conscious associative links between the obstacle and the behavior instrumental to overcoming the obstacle thereby fostering goal pursuit, it might be useful to add a strategy that strengthens these associative links even further. Forming implementation intentions qualifies as such a strategy. Accordingly, MC and II were combined into one strategy called mental contrasting with implementation intentions (MCII; Oettingen and Gollwitzer, 2010). As described above, mental contrasting instigates goal pursuit (goal commitment and goal striving), and goal commitment is a prerequisite for the beneficial effects of implementation intentions (Sheeran et al., 2005). Mental contrasting also helps to identify the critical situation for the if-part (obstacle) of implementation intentions and the instrumental action for the then-part of the plan (overcoming the obstacle).

Numerous studies have demonstrated the effectiveness of MCII as an intervention, many of them in the health domain. Participants who employed MCII to eat more healthily engaged in more physical exercise over a period of 4 months (Stadler et al., 2009), consumed more fruits and vegetables over a period of 2 years (Stadler et al., 2010), and ate less red meat over 5 weeks (Loy et al., 2016). MCII helped increase physical exercise and weight reduction in patients who had a stroke over a period of 1 year (Marquardt et al., 2017), and increased physical capacity in patients with chronic back pain over 3 months (Christiansen et al., 2010). An intervention study by Sailer et al. (2015) observed that MCII even helped patients with schizophrenia in autonomy-focused clinical hospital settings to translate their exercising intentions into action.

When applied to the domain of interpersonal relationships, MCII increased commitment to the relationship and decreased insecurity-related behaviors such as avoiding sensitive topics (Houssais et al., 2013). MCII also helps people to manage their time. For example, it supported both working mothers from low-income backgrounds who were enrolled in vocational education and medical residents who were serving in intensive care units to find the time to study for their exams (Oettingen et al., 2015; Saddawi-Konefka et al., 2017). Regarding MCII interventions taught online, Kizilcec and Cohen (2017) found in two studies that MCII delivered as an 8-min online intervention in the context of massive open online courses (MOOC) substantially increased completion rates of the online courses.

## The Present Research

We invited nurses from all over Germany to participate in our online study to insure that institutions with different kinds of organizational and hierarchical structures were covered. Based on the results of past research on behavior change via MCII described above, we hypothesized that MCII should help nurses to fulfill their wishes regarding reducing their stress, whether the specific content of the wish pertains to health, interpersonal relations, or achievement. We tested the effectiveness of engaging in MCII (to be performed over 3 weeks on a daily basis) compared to a no-treatment control group that only was asked to explore their wishes regarding achieving less stress. We also added a further intervention group (i.e., IIMCII); here participants were asked to furnish the goal to engage in daily MCII exercises, assigned by the experimenter, with an implementation intention that specified when and where they planned to execute these MCII exercises. We included this third condition because we were worried that healthcare professionals might not find the time to perform MCII on a regular basis, and therefore only a moderate stress reduction might be observed in the mere MCII group. Based on previous research showing beneficial effects of implementation intentions on goal attainment (Gollwitzer, 2014), we hypothesized that participants in the IIMCII condition would benefit from using if-then plans that specify the situation in which they wanted to engage in MCII, thus showing even more stress reduction and even more work engagement than participants in the mere MCII condition. Participants’ stress level was assessed prior to the intervention and 3 weeks later with established self-report questionnaires pertaining to perceived stress, stress-related physical symptoms, and work engagement.

## Materials and Methods

### Design

The study used a randomized 3 (Intervention, between: control vs. MCII vs. IIMCII) × 2 (Time, within: baseline-measurement vs. post-treatment measurement) factorial design. As dependent variables we assessed (a) perceived stress, (b) current physical symptoms, and (c) work engagement, once at the beginning of the interventions and then again 3 weeks later.

### Participants

**FIGURE 1:**
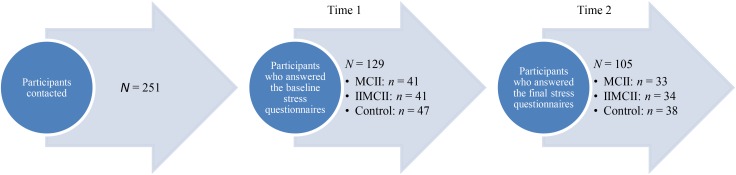
Flowchart of the participants.

The administrations of health care institutions all over Germany were contacted via regular mail, emails, and phone calls over a time period of 3 months (October to December). The administrators were asked to pass on a prepared email message to their nurses, asking them whether they would be interested in participating in a study on stress reduction. This message explained that participation would potentially help reduce one’s stress level, and that there is a chance (albeit small) of winning a gift certificate of 100 Euro. The message also contained the email address of the experimenter whom the nurses should contact if they wanted to register for the study. Those who registered (*N* = 251 nurses) were contacted in return by the experimenter (again via email) and given access to the study website that had been created by using the *soscisurvey.de* data collection service. Participants who entered the website (*N* = 129) were randomly assigned to the three conditions of the study (MCII = 41, and IIMCII = 41, Control = 47) (see **Figure 1**).

On the study website, all participants were first asked to complete three questionnaires assessing their baseline stress level. Thereafter, they were guided to the instructions of the respective experimental condition. Three weeks later, the experimenter contacted the participants via email encouraging them to re-enter the study website at *soscisurvey.de.* This time they were only asked to fill out the three stress-related questionnaires a second time. Thirty-three participants of the MCII group, 34 participants of the IIMCII group, and 38 participants of the control group answered the final stress questionnaires: a return rate of 80.5, 82.9, and 80.9%, respectively. These 105 participants (82% female) had a mean age of 40.22 years (*SD* = 10.18), and an average length of work experience of 17.60 years with a minimum of 0 years (in training) and a maximum of 40 years. Eighty-one of the participants worked in a hospital, 5 in residential care, 5 in a nursing home, 5 in psychiatric institutions, 3 in day hospitals, 2 in rehabilitation centers, and 4 in not specified institutions. Also, 70.5% had full-time positions; 54.3% worked in shifts, 40.0% in night shifts, and 48.6% claimed to irregularly work extra hours.

### Materials and Procedure

#### Baseline Measurement

At Time 1, all participants had to first fill out three established questionnaires. Two of them focused on assessing participants’ overall stress level, one of them by targeting participants’ perceived stress and the other by obtaining information on physical symptoms of stress. Perceived stress and physical symptoms in response to a stressor both pertain to people’s overall stress level (Folkman and Lazarus, 1990). In addition, we used a questionnaire pertaining to a potential consequence of having a lot of stress: participants’ level of work engagement. The order of the questionnaires was as follows: perceived stress, work engagement, and physical symptoms of stress. We placed the work engagement questionnaire in between the two stress questionnaires so that participants’ answers to the perceived stress questionnaire would not carry over to reporting on physical symptoms of stress.

#### Perceived Stress

Perceived stress was measured with an adapted version of the Perceived Stress Questionnaire-20 (PSQ-20; Fliege et al., 2001, 2005). The 20-item questionnaire asks participants to base their responses on the last 3 weeks: “Please specify how often the following statements applied to you in the last 3 weeks.” Sample items: *You felt rested* (reverse coded), *You had problems relaxing*, *You felt safe and protected* (reverse coded), and *You were under time pressure*. Responses ranged from 1 = *almost never* to 4 = *usually*. This questionnaire covers the subjective experience of stress independent of specific stressors on four subscales with five items each. The subscale *worries* targets current worries, anxiety about the future and emotions of frustration; the subscale *tension* pertains to current fatigue, imbalance, and the lack of physical relaxation; the subscale *joy* covers positive feelings; and the subscale *demands* measures current lack of time, time pressure, or burden of tasks. The first three scales target the internal stress reactions of an individual, while the scale *demands* assesses the perception of external stressors. The PSQ has been demonstrated to be reliable and valid. Fliege et al. (2001, 2005) report that the questionnaire has high internal consistency (Cronbach’s α = 0.85). In our study, the baseline perceived stress questionnaire had a good internal consistency as well (Cronbach’s α = 0.93); composite reliability (CR) was 0.76.

##### Physical symptoms

Symptoms were measured with the physical symptoms subscale of the Burnout Screening Scales II inventory (BOSS II; Hagemann and Geuenich, 2009). This subscale targets the physical symptoms of individuals, especially as they would typically occur in people with a burnout syndrome or with chronic stress. It contains ten items that cover various physical disabilities, pains, and somatic ailments. The emphasis of this scale is placed on cardiovascular complaints. Further, functional constraints of the respiratory, digestion, and immune systems as well as general parameters of states of stress and sleeping quality were assessed. We instructed participants by stating: “Please specify whether the following statements applied to you in the last 3 weeks.” Examples were: *I feel pressure in my chest*, *I am plagued by serious headaches*, and *I have respiratory difficulties*. Responses ranged from 1 = *does not apply* to 6 = *strongly applies*. The Burnout Screening Scales (BOSS II) inventory has been demonstrated to be reliable and valid (Hagemann and Geuenich, 2009). In our sample the internal consistency at the baseline assessment was also high, Cronbach’s α = 0.83; composite reliability (CR) was 0.76.

##### Work engagement

We assessed work engagement as an indirect indicator of stress assuming that stressed out nurses show reduced work engagement. Work engagement was assessed using a condensed version of the Utrecht Work Engagement Scale (UWES-9; Schaufeli et al., 2006). The UWES-9 questionnaire measures work engagement with three subscales: *vigor* in the sense of energy, strength and perseverance at work, *dedication* in the sense of interest, inspiration, pride, and challenge, and *absorption* in the sense of concentration at work, loss of a sense of time, and felt importance of work. The instructions for this questionnaire were: “Please choose the respective answer that best applies to you regarding the last 3 weeks.” Sample items are: *My work inspires me*, *My work is fulfilling*, and *I am proud of my wor*k. The answer scale ranged from 1 = *never*, 2 = *almost never*, 3 = *occasionally*, 4 = *regularly*, 5 = often, 6 = *very often*, to 7 = *always*. Schaufeli et al. (2006) found a good internal consistency of the questionnaire (Cronbach’s α = 0.80). In our study, the UWES-9 assessed at baseline had a high internal consistency as well (Cronbach’s α = 0.94); composite reliability (CR) was 0.95.

#### Instructions to Use Stress-Reduction Strategies

**FIGURE 2:**
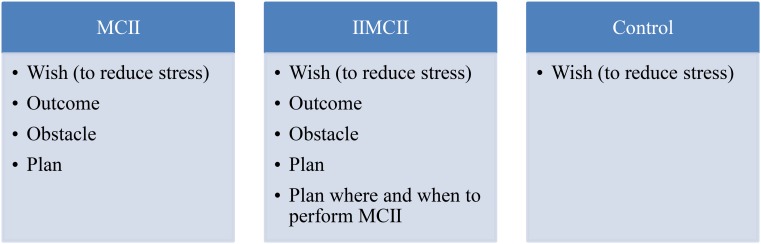
Schematic overview of the intervention instructions provided in the three conditions: MCII, IIMCII, and control.

**FIGURE 3:**
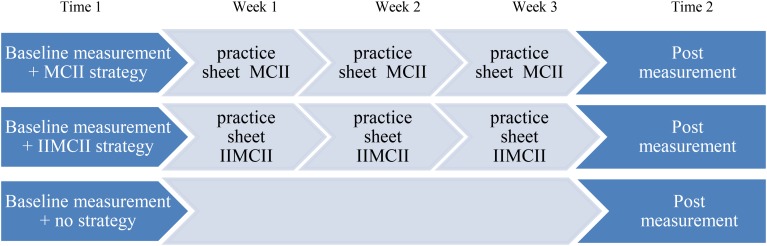
Time line of the course of events in the three conditions: MCII, IIMCII, and control.

After having filled out the three questionnaires, participants of all conditions read instructions asking them to first think about the topic of stress at work and to answer three general questions about stress (see **Figures 2**, **3**). Participants read:

Less Stress at Work – A Dream? Make this dream become a reality! As a start, please think about stress. Please answer the following questions (take as much time as you need).
What does stress mean for me?
What does less stress mean for me?
Why do I want less stress?
My wish for less stress: …
Please write down your most important personal wish for less stress at your work place in the next 3 weeks in one or two sentences. What exactly do you want?

##### MCII group

Thereafter, MCII-participants were asked to go through the following mental exercise at their own pace. First, they had to note the best possible outcome of their wish for less stress. Afterward, they were asked to imagine the events and experiences associated with this best outcome. They subsequently had to write down their thoughts. The exact instructions read:

Please think about the best outcome of fulfilling your wish for less stress and take note of your thoughts by writing down one or two sentences.

(1) The best outcome of fulfilling my wish in the next 3 weeks is: …
(2) Now imagine the best outcome in your thoughts. Imagine the events and experiences you associate with the best outcome. Please take as much time as you need to imagine it as fully as you can.
(3) Now please write down all the thoughts and images you had regarding the best outcome: …

Then, the main obstacle had to be identified and elaborated. The participants were asked to consider what speaks against the fulfillment of the wish in the next 3 weeks. They had to imagine the events and experiences related to this main obstacle separating them from reaching their goal. Then, they had to take note of this main obstacle and vividly imagine it. The instructions stated:

Sometimes, our wishes are not fulfilled. Think about what speaks against your wish being fulfilled in the next 3 weeks.

(1) What is your main obstacle? Please write it down in one or two sentences.
(2) My main obstacle to fulfilling my wish in the next 3 weeks is:…
(3) Now please imagine this main obstacle. What are the events and experiences that could hold you back? Please take as much time as you need to imagine it as fully as you can.
(4) Now please write down all the thoughts and images you had regarding your main obstacle:…

Finally, the participants were introduced to the forming of an if-then plan. The participants had to make their own if-then plan by transferring their obstacle to the if-part and the wish-fulfilling action to the then-part of the plan. Afterward, they were instructed to repeat the if-then plan several times. Instructions read:

Please think about how you could act to overcome or prevent your obstacle in the next weeks. How can you act to fulfill your wish? Please briefly describe this wish-fulfilling action. My action to fulfill my wish in the next weeks by overcoming my obstacle is:…
An important tool for your stress management strategy is the forming of if-then plans. If-then plans link your obstacle with an action that is instrumental to realizing your wish. They have the following format:
***If*** *the obstacle arises*, ***then*** *I will execute the following wish-fulfilling action!*
Please formulate your own if-then plan now by linking your previously mentioned obstacle with your chosen wish-fulfilling action:

$$If...\frac{}{\left[\text{Please enter your previously mentioned obstacle here}\right]}occurs,$$

$$\text{then I will}...\frac{}{\left[\text{Please enter the previously mentioned wish-fulfilling acyion here}\right]}!$$

Now imagine this if-then plan and go through it in your mind!

The participants were then instructed to perform this four step strategy in their mind on a daily basis at work for the following 3 weeks. To facilitate this, the MCII strategy was summarized in the form of four questions:

What is the best possible outcome today of my wish to have less stress?
What is the main obstacle today to fulfilling this wish?
How can I act to overcome this obstacle?
What is my if-then plan today?

Finally, participants were asked to think about a calm situation and quiet moment in their daily work that would be ideal for them to go through this exercise in their mind. The participants were then asked to set a personal goal for the following 3 weeks: “I will use the stress management strategy once a day!”

To summarize how MCII is done, participants received a practice sheet listing the four steps of the MCII exercise. This practice sheet was sent to them again via email by the experimenter twice, once after 1 week and then after 2 weeks.

##### IIMCII group

The instructions given to the IIMCII participants was identical to those received by the MCII participants with the following difference: The participants in the IIMCII condition were not only assigned to set a goal of using MCII every day for the next 3 weeks (i.e., “I will use the stress management strategy once a day!”) but in addition to make an if-then plan in their mind that specified when and where the daily exercise should take place. The instructions for making this plan read:

Please write down a calm situation and time most suitable to performing this exercise:

$$\frac{}{\left[\text{Please enter the chosen situation and time}\right]}$$

Now make an if-then plan in which you specify that you will perform this exercise once a day in this calm situation and time over the next 3 weeks:

$$If\frac{}{\left[\text{Please enter the chosen situation and time}\right]}\text{occurs, then I will do this exercise!}$$

Please repeat this if-then plan several times in your mind.

As with the MCII participants, the IIMCII participants then also received a practice sheet listing the four steps of MCII. This practice sheet was also sent to IIMCII participants a week as well as 2 weeks later.

##### Control group

The participants of the control group were only requested to answer the following three questions: What does stress mean for me? What does less stress mean for me? Why do I want less stress? In addition, they had to specify a personal wish with respect to achieving less stress at their work place in the next 3 weeks, and what exactly that wish implied.

#### Assessment of the Dependent Variables

Three weeks later, participants of all three conditions received an email from the experimenter with a link to the follow-up website at *soscisurvey.de.* When opening this website, participants received instructions to fill out the three questionnaires used at baseline within the next couple of days. The questionnaires were presented in the order used at baseline: perceived stress, work engagement, and then physical symptoms. Again, the internal consistency for each of the three questionnaires was high; Cronbach’s α of 0.95, 0.95, and 0.85, respectively, were observed; composite reliability (CR) was 0.86, 0.96, and 0.78.

## Results

### Descriptive Analyses

As perceived stress and physical symptoms both speak directly to the participants’ stress level, we *z*-transformed and combined the 20 items of the PSQ-20 and the 10 items of the BOSS II to an overall stress index. Internal consistency was high at both T1, Cronbach’s α = 0.93, and T2, Cronbach’s α = 0.95; composite reliability (CR) was 0.78 at T1 and 0.83 at T2. At Time 1, overall stress and work engagement did not differ across conditions, *p*s > 0.14 (see **Table 1** for means and standard deviations of all dependent variables).

**Table 1:** Means (standard deviations) of the dependent variables.

		Overall stress		Work engagement	
			
		Time 1	Time 2	Time 1	Time 2
Overall		0.02 (0.58)	0.00 (0.64)	4.23 (1.20)	4.24 (1.26)
	Control	0.16 (0.65)	0.22 (0.73)	4.06 (1.23)	4.03 (1.40)
	IIMCII	-0.04 (0.41)	-0.05 (0.46)	4.22 (1.18)	4.11 (1.01)
	MCII	-0.09 (0.61)	-0.20 (0.63)	4.43 (1.21)	4.63 (1.27)


### Change of Overall Stress

#### Statistical Analysis

An analysis of covariance was used to assess whether participants of the MCII group or the IIMCII group show less stress after 3 weeks than participants of the control group. Change of overall stress was analyzed with a univariate ANCOVA, adjusting for overall stress at Time 1. We followed up this ANCOVA with two-tailed *post hoc* pairwise comparisons testing for differences between the MCII group and the control group as well as the IIMCII group, and between the IIMCII and the control group. Due to the dropout rate we then performed an intention to treat analysis with all 129 participants who answered the baseline questionnaire. The missing values of 24 participants at Time 2 were replaced by their respective baseline value.

#### Statistical Results

**FIGURE 4:**
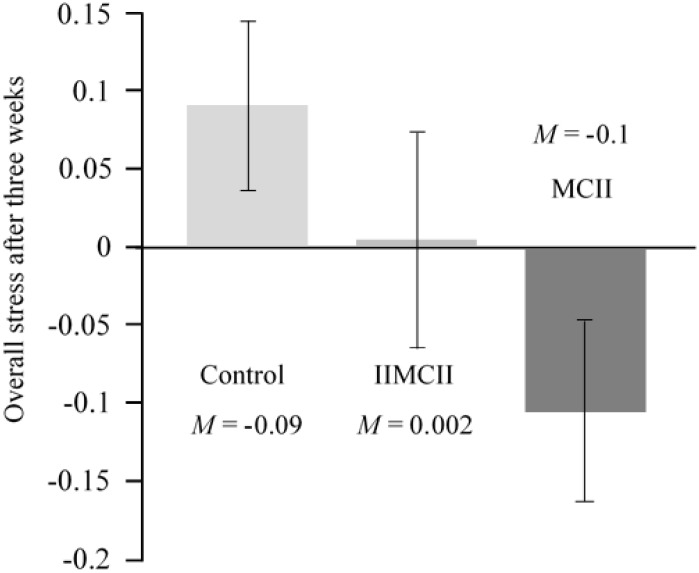
Overall stress after 3 weeks assessed by the Perceived Stress Questionnaire (PSQ-20) and the Burnout Screening Scales II inventory (BOSS II) in the three groups of the intervention study, controlling for baseline scores.

We observed an almost significant difference between conditions with respect to the change in overall stress, *F*(2,101) = 2.85, *p* = 0.062, $\eta_{p}^{2}$ = 0.053. Pairwise comparisons revealed less stress in the MCII group as compared to the control group, *t*(101) = 2.39, *p* = 0.019, $\eta_{p}^{2}$ = 0.053, 95% CI [-0.348, -0.032]. We neither found a difference between the MCII group and the IIMCII group, *t*(101) = 1.30, *p* = 0.198, $\eta_{p}^{2}$ = 0.016, 95% CI [-0.264, 0.055] nor between the IIMCII and the control group, *t*(101) = 1.09, *p* = 0.277, $\eta_{p}^{2}$ = 0.012, 95% CI [-0.242, 0.070] (see **Table 2** and **Figure 4**). Bonferroni-Holm adjustment of the *p*-values yielded the following results: *p* = 0.057, *p* = 0.396, and *p* = 0.396, respectively.

**Table 2:** Comparing the three conditions regarding overall stress and work engagement.

	*F*	*p*	$\eta_{p}^{2}$		*t*	*p*	$\eta_{p}^{2}$	95% CI	
Overall stress	2.85	0.062	0.053						
				MCII vs. Control	2.39	0.019	0.053	-0.348	-0.032
				MCII vs. IIMCII	1.30	0.198	0.016	-0.264	0.055
				IIMCII vs. Control	1.09	0.277	0.012	-0.242	0.070
Work engagement	2.98	0.055	0.056						
				MCII vs. Control	2.02	0.046	0.039	0.006	0.564
				MCII vs. IIMCII	2.22	0.029	0.047	0.034	0.601
				IIMCII vs. Control	0.23	0.816	0.001	-0.309	0.244


In our intention to treat analysis, we observed the same pattern of results between conditions with respect to change in overall stress, *F*(2,125) = 2.87, *p* = 0.060, $\eta_{p}^{2}$ = 0.044. Again, pairwise comparisons revealed less stress in the MCII group as compared to the control group, *t*(125) = 2.39, *p* = 0.018, $\eta_{p}^{2}$ = 0.044, 95% CI [-0.285, -0.027]. Again, we neither found a difference between the MCII group and the IIMCII group, *t*(125) = 1.32, *p* = 0.189, $\eta_{p}^{2}$ = 0.014, 95% CI [-0.219, 0.044] nor between the IIMCII and the control group, *t*(125) = 1.06, *p* = 0.292, $\eta_{p}^{2}$ = 0.009, 95% CI [-0.196, 0.059]. Bonferroni–Holm adjustment of the *p*-values yielded the following results: *p* = 0.054, *p* = 0.378, and *p* = 0.378, respectively.

### Change of Work Engagement

#### Statistical Analysis

Work engagement is often influenced by working conditions. To adjust for different working conditions, we coded reported working condition with 0 = *no shift work*, 1 = *either shift work or night shifts*, and 2 = *shift work as well as night shifts*. Working condition correlated significantly with change of work engagement, *r* = 0.20, *p* < 0.05. To control for the influences of unfavorable working conditions on work engagement (Sonnentag, 2003), working condition was incorporated in the analysis as a covariate. Change of work engagement was analyzed with a univariate ANCOVA, adjusting for work engagement at Time 1 and working condition. As we have done in our analysis regarding change in overall stress, we followed up this ANCOVA with two-tailed *post hoc* pairwise comparisons testing for differences between the MCII group and the control group as well as the IIMCII group, and between the IIMCII and the control group. Finally, we conducted the intention to treat analysis just like we have done it with respect to change in overall stress.

#### Statistical Results

**FIGURE 5:**
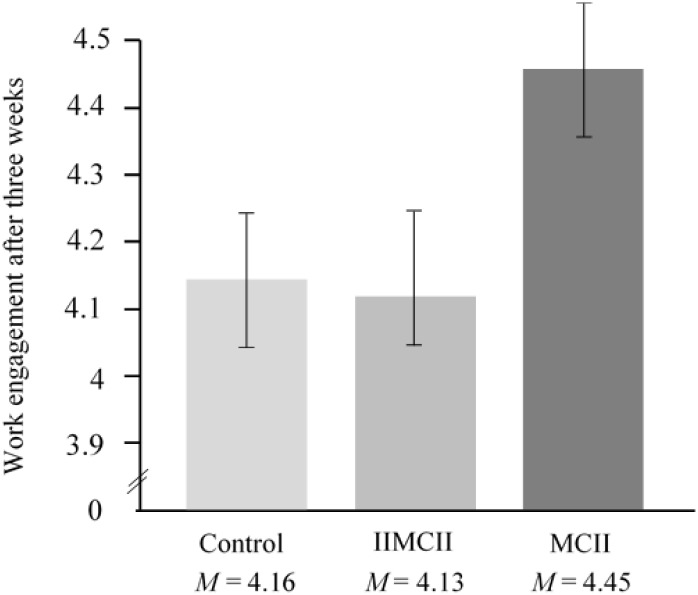
Work engagement after 3 weeks assessed by a questionnaire from Schaufeli et al. (2006) in the three groups of the intervention study, controlling for baseline scores.

We found an almost significant difference in work engagement, *F*(2,100) = 2.98, *p* = 0.055, $\eta_{p}^{2}$ = 0.056 between conditions. Pairwise comparisons revealed (1) more work engagement in the MCII group than in the IIMCII group, *t*(100) = 2.22, *p* = 0.029, $\eta_{p}^{2}$ = 0.047, 95% CI [0.034, 0.601], as well as (2) in the control group, *t*(100) = 2.02, *p* = 0.046, $\eta_{p}^{2}$ = 0.039, 95% CI [0.006, 0.564]. As with overall stress, we found no difference between the IIMCII group and the control group, *t*(100) = 0.23, *p* = 0.816, $\eta_{p}^{2}$ = 0.001, 95% CI [-0.309, 0.244] (see **Table 2** and **Figure 5**). Bonferroni–Holm adjustment of the *p*-values yielded the following results: *p* = 0.087, *p* = 0.092, and *p* = 0.816, respectively.

The intention to treat analysis showed the same difference between conditions with respect to change in work engagement, *F*(2,124) = 3.24, *p* = 0.042, $\eta_{p}^{2}$ = 0.050. Pairwise comparisons revealed more work engagement in the MCII group as compared to the IIMCII group, *t*(124) = 2.33, *p* = 0.021, $\eta_{p}^{2}$ = 0.042, 95% CI [0.041, 0.504] and to the control group, *t*(124) = 2.09, *p* = 0.039, $\eta_{p}^{2}$ = 0.034, 95% CI [0.012, 0.466]. We did not find a difference between the IIMCII and the control group, *t*(124) = 0.29, *p* = 0.770, $\eta_{p}^{2}$ = 0.001, 95% CI [-0.258, 0.191]. Bonferroni–Holm adjustment of the *p*-values yielded the following results: *p* = 0.063, *p* = 0.078, and *p* = 0.770, respectively.

## Discussion and Outlook

The MCII intervention reduced the participating nurses’ overall stress and improved work engagement as compared to a no-treatment control group. Even though we measured stress reduction and work engagement after a relatively short period of time (i.e., already after 3 weeks), we observed improvements on both of these variables measured reliably with established questionnaires covering the different aspects of perceived stress (i.e., worries, tension, reduced joy, heightened demands as well as typical physical symptoms such as headaches, back pain) and work engagement (i.e., vigor, dedication, and absorption).

We did not give the nurses hints on what they can do to reduce their worries, tensions, demands, and headaches, and what can be done to increase vigor, dedication, and absorption with their work. Rather, we left it up to the nurses to detect their stress-reducing wish and best outcome, and to identify what hinders them to realize their wishes for less stress at the work place, and what they want to do to overcome these obstacles. Also, we did not send daily reminders to the nurses to perform the MCII exercise. We just told them at the end of teaching the exercise to perform MCII on a daily basis. That is, the whole intervention encompassed one session at the outset in which the participants had to go through the MCII exercise in writing. From then on, the participants were on their own, engaging in the MCII exercise in their mind over the subsequent 3 weeks.

In order to ensure high fidelity, our instructions in the three conditions were adapted from past MCII intervention research (e.g., summary by Oettingen and Gollwitzer, 2010; Oettingen, 2012, 2014). These intervention studies pertained to a variety of samples reaching from children at risk for ADHD to healthy adults and to patients recovering from stroke (Marquardt et al., 2017). In addition, our participants received three practice sheets that depicted the steps of the intervention in the respective condition (**Figures 2**, **3**). The first practice sheet was provided right after the intervention, the other two 1 and 2 weeks later. This way we tried to ensure that participants closely followed the instructions they had received in the intervention session. The only additional information they received were two emails from the experimenter, 1 and 2 weeks after the training session, containing a practice sheet depicting the four steps of MCII.

The IIMCII group did not show the hypothesized pattern of results: It did not show the expected heightened reduction in stress level and it did not show the enhanced work engagement as compared to the MCII group; not even differences to the control group emerged. We established the IIMCII group as we were worried that healthcare providers simply may be too overburdened in their daily work to act on the assigned goal to engage in MCII on a regular basis. Simply thinking of a quiet time and place in which the MCII exercise could be performed may not suffice. Rather, it might need an additional implementation intention that specifies this critical time and place in the if-part of the plan. After all, implementation intentions have been found to help people remember to act on their goals, in particular when they suffer from high cognitive load (e.g., Cohen and Gollwitzer, 2008). So why did we not find the expected effects on stress reduction and heightened work engagement in the IIMCII group?

Three answers come to mind: First, asking IIMCII participants right after the training session has ended to make an if-then plan at which time and place they want to engage in the daily MCII exercise may have limited IIMCII participants to this very time and place for the rest of the 3 weeks. In contrast, the MCII group only specified the goal to use MCII on a daily basis at a quiet time and place. This should allow for more flexibility in case unexpected or even better opportunities for performing MCII open up (i.e., more appropriate quiet times and places). Second, action control by implementation intentions is known to be characterized by features of automaticity (i.e., it is fast, runs off outside of awareness, and is effortless). Given that mental contrasting is an effortful cognitive procedure that requires slowness and imagination, the mindset associated with acting on the implementation intention to use a pre-specified time and place may undermine the effortful cognitive procedure of mental contrasting. Third, and most importantly, we cannot assume that participants had a high commitment to use the MCII exercise on a daily basis. They have heard about it for the first time just before – when they had started the experiment – and thus they might have not yet fully trusted its effects. Having no strong commitment to use MCII on a daily basis should attenuate the effectiveness of forming implementation intentions created in the service of using MCII. As described before, the prerequisite of implementation intentions is the commitment for the overarching goal (Sheeran et al., 2005).

### Strengths and Weaknesses

This procedure is more parsimonious than classic stress interventions. The latter focus on intensively training participants in performing specific stress reduction strategies (e.g., emotion control strategies such as distancing oneself from an overly arousing event, conflict resolution strategies, and relaxation techniques), and this is commonly done via close supervision by the interventionist. Accordingly, the present study could have added a further control group that used a typical classic stress intervention (e.g., directing attention away from work problems). It would have been interesting to see whether the time and cost effective MCII exercise lives up to the success of classic intervention programs. Also, from a methodological point of view the present study would have benefited from assessing data on participants’ adherence to the MCII instructions and the frequency and context of participants using MCII. How often did the participants in the MCII and IIMCII perform the MCII exercise, and did the frequency of performing it correlate positively with stress reduction? Future research will have to make the necessary changes to the design (add a further control group) and assessment (add a measure of degree of adherence).

### Future Research

Future research may address the question of how MCII effects can be strengthened even further. One way of doing so pertains to combining MCII with other types of interventions. For instance, it might be worthwhile to add a self-affirmation exercise (Harris et al., 2014) or a mindfulness exercise (Smith, 2014) prior to having participants perform MCII. Both of these exercises are known to reduce self-defensiveness and thus it seems possible that participants, in the aftermath of these exercises, find it easier to detect truly personal wishes, outcomes, and obstacles when engaging in MCII, which in turn should make MCII more effective.

Second, future research may address the question of the content area for which MCII is taught and practiced. Our MCII exercise solely focused on wishes reducing stress at the work place (though we do not know whether participants had used MCII for other wishes as well). Our teaching did not explicitly include wishes on how to recover from work stress at home and during leisure time. It is, however, a person’s work-life balance that is increasingly recognized as an important factor for living a physically healthy life (Sonnentag, 2003). Future research may ask participants to perform MCII on integrative wishes regarding work-life balance.

Third, we did not provide information on effective stress-reduction strategies as discovered in stress research (e.g., effective emotion regulation strategies at work or relaxation techniques at home). Such a combination of providing relevant information first before participants engage in the MCII exercise has been used effectively in changing people’s eating behavior and making people more physically active (e.g., Stadler et al., 2009, 2010; Marquardt et al., 2017). Future research may extend the focus of MCII interventions regarding the work place to relaxation after work, and by adding an information session on how to best reduce stress at work and at home prior to asking participants to engage in the MCII exercise.

Fourth, it might be valuable to learn more about the nurses’ stress-relevant personal attributes before the MCII instructions are given. For instance, if one nurse is suffering from emotion control problems whereas another is overly impulsive, knowing about this by handing out relevant personality questionnaires at the outset of the study would allow gearing their wishes to more effectively cope with these shortcomings. Recent research shows that MCII can indeed be targeted toward affective aspects of one’s outcome and obstacle (Ruissen et al., 2018) or toward enhancing reflection over impulsivity (Bieleke et al., 2017).

And finally, there remains the question of how one can make MCII effects long-lasting. One approach pertains to going beyond helping the nurses to individually cope with their stressful situations but encouraging them to get involved with efforts to change the team or organization they are working for into a less stressful one. This may often be not possible, but if successful it heightens the chances that the reduced level of personal stress the nurses have achieved via improved self-regulation will stay stable over time (Heaney et al., 1995).

A more practical and feasible route to achieving long-lasting stress reduction, however, might be to turn the use of MCII into a habit. Anecdotally, we find that using MCII by facilitating behavior change is rewarding. If so, open access to guidelines of how to use MCII during daily life might be a first step to establish the habitual use of MCII. Indeed, the guidelines of how to use MCII – which colloquially has been given the acronym of WOOP for Wish, Outcome, Obstacle, Plan – is available to the public on a website^1^ and in an app called WOOP.

## Ethics Statement

This study was carried out in accordance with the recommendations of the ethics committee of the University of Konstanz. All subjects gave written informed consent in accordance with the Declaration of Helsinki.

## Author Contributions

PG, GO, and CF jointly designed the study and CF conducted it. DM analyzed the data and prepared the presentation of the results. All authors worked on preparing a first draft and on revising the manuscript into its final form.

## Conflict of Interest Statement

The authors declare that the research was conducted in the absence of any commercial or financial relationships that could be construed as a potential conflict of interest.

## Footnotes

1 www.woopmylife.org
